# Is It Human or Animal? The Origin of Pathogenic *E. coli* in the Drinking Water of a Low-Income Urban Community in Bangladesh

**DOI:** 10.3390/tropicalmed6040181

**Published:** 2021-10-05

**Authors:** Jannatul Ferdous, Ridwan Bin Rashid, Rebeca Sultana, Sabera Saima, Musharrat Jahan Prima, Anowara Begum, Peter Kjær Mackie Jensen

**Affiliations:** 1 Department of Microbiology, University of Dhaka, Dhaka 1000, Bangladesh; ridwanrashidunivdhaka@gmail.com (R.B.R.); saberasaima123@gmail.com (S.S.); prima.musharrat@gmail.com (M.J.P.); anowara@du.ac.bd (A.B.); 2 Copenhagen Center for Disaster Research, Section for Global Health, Department of Public Health, University of Copenhagen, 1014 Copenhagen, Denmark; rebeca@icddrb.org (R.S.); mackie@sund.ku.dk (P.K.M.J.); 3 Institute of Health Economics, University of Dhaka, Dhaka 1000, Bangladesh; 4 International Centre for Diarrhoeal Disease Research, Dhaka 1000, Bangladesh

**Keywords:** diarrhea, *Escherichia coli* pathotypes, drinking water, one health, ETEC, phylogenetic

## Abstract

This study aimed to investigate the origin of diverse pathotypes of *E. coli*, isolated from communal water sources and from the actual drinking water vessel at the point-of-drinking inside households in a low-income urban community in Arichpur, Dhaka, Bangladesh, using a polymerase chain reaction (PCR). Forty-six percent (57/125, CI 95%: 41−58) of the isolates in the point-of-drinking water and 53% (55/103, CI 95%: 45−64) of the isolates in the source water were diarrheagenic *E. coli*. Among the pathotypes, enterotoxigenic *E. coli* (ETEC) was the most common, 81% (46/57) of ETEC was found in the point-of-drinking water and 87% (48/55) was found in the communal source water. Phylogenetic group B1, which is predominant in animals, was the most frequently found isolate in both the point-of-drinking water (50%, 91/181) and in the source (50%, 89/180) water. The phylogenetic subgroup B2_3_, usually of human origin, was more common in the point-of-drinking water (65%, 13/20) than in the source water (35%, 7/20). Our findings suggest that non-human mammals and birds played a vital role in fecal contamination for both the source and point-of-drinking water. Addressing human sanitation without a consideration of fecal contamination from livestock sources will not be enough to prevent drinking-water contamination and thus will persist as a greater contributor to diarrheal pathogens.

## 1. Introduction

*Escherichia coli* is an inhabitant of the mammalian colon and has for decades been used as the global indicator for the possible presence of fecal pathogens in food and especially drinking water. However, not all *E. coli* are created equal. While some *E. coli* types are harmless, other variants are highly pathogenic and capable of causing severe diarrheal diseases with significant morbidity and mortality [1]. The Global Burden of Disease study in 2015 included two of the six major diarrheagenic *E. coli* (DEC) strains in the top 10 of diarrhea-causing agents worldwide: enterotoxigenic *E. coli* (ETEC) with 51,186 (26757–83064) annual deaths and 223 million cases (145 million–323 million) enteropathogenic *E. coli* (EPEC) with 12,337 (4439–25697) annual deaths and 14 million cases (6 million–31 million) [2]. The other DEC are: enterohemorrhagic *E. coli* (EHEC), enteroinvasive *E. coli* (EIEC), enteroaggregative *E. coli* (EAEC), and diffusely adhering *E. coli* (DAEC) [3]. 

The six DEC strains do not only differ in their contribution to the global disease burden but they also significantly differ in their infectious dose, from >10^6^ organisms for a laboratory-grown ETEC to a single EHEC cell needed to cause disease [4,5]. Despite differences in infectious doses, the interventions to mitigate DEC and other diarrhoeal diseases have traditionally relied on breaking the fecal-oral transmission routes, as illustrated in the F-diagram [6]. The interventions have been a combination of clean WAter, improved Sanitation, and Hygiene (WASH), of which the sanitation intervention is of primary importance as the excreta would be in a hygienic confined space (toilet/latrine) without the possibility of being transmitted via the other routes. However, the authors have argued that the ’A’ in WASH should represent Animals, hereby acknowledging that *E. coli* can have a human or animal origin. Putting the ’A’ into WASH has huge implications for the intervention strategy, as the focus would shift from traditional public health (human sanitation interventions) to a more holistic One Health approach (excreta management from domestic animals) [7]. However, to investigate the need for a possible change of focus within WASH interventions, the magnitude of animal transmitted *E. coli* as compared to human *E. coli* needs to be established, and a method is required to differentiate between human and animal *E. coli*. 

To identify the origin of fecal contamination in public and private water sources, Bacteroidales have been utilized to track the microbiological source [8]. Bacteroidales have features that demonstrate that this order of bacteria can be of use for Microbial Source Tracking (MST): they are obligate anaerobes and hence cannot replicate outside the gut [9], they demonstrate host specificity [10,11], and they are present in feces in higher numbers. Although there are merits of using Bacteroidales for MST, human-associated Bacteroidales assays were found to have a low sensitivity and moderate to high cross-reactivity with dog (20 to 80%) and chicken fecal samples (60 to 100%) [12]. The ruminant-cattle-associated assays also cross-reacted with dog and chicken samples [12]. Therefore, due to the cross-reactivity within various host species, it is difficult to identify the fecal origin of contamination (animal or human) using this MST technique. 

Alternatively, the use of phylo-grouping of *E. coli* from fecal sources has been demonstrated as a complementary tool to track the major sources of fecal contamination i.e., animal or human in environmental water samples (rivers, recreational waters, agricultural waters, and sewage waters) [13,14,15,16]. These studies have demonstrated that the distribution of the phylogenetic subgroup, group, and genetic markers are not randomly distributed among the hosts analyzed. The phylogroups of *E. coli* usually differ in their ecological niches, life history, and characteristics, such as their ability to exploit different sugar sources, antibiotic-resistance profiles, and growth rates [17]. This in turn reflects the dietary source of the host species and the antibiotics provided in their food intake [18,19]. 

*E. coli* strains can be assigned to one of the main phylogenetic groups: A, B1, B2, or D based on the genetic markers *chuA*, *yjaA,* and the DNA fragment TspE4.C2 [20]. Escobar-Páramo et al. (2006) and Carlos et al. (2010) purposefully selected animals and human subjects, and isolated *E. coli* from their feces to show the prevalence of specific phylotypes in specific host species [14,15]. Escobar-Páramo et al. (2006) phylo-grouped 1898 *E. coli* isolates originating from 387 animals (birds and mammals) and 760 human isolates originating from 152 healthy humans [15]. The prevalence of the *E. coli* phylogenetic groups was different in birds, non-human mammals, and humans with a predominance of D/B1, A/B1, and A/B2 strains, respectively (Table 1). The repartition within the four major phylogenetic groups (A, B1, D, B2) of the *E. coli* strains depends on the population structure, which is shaped by the habitat, dietary pattern, climate, and time. For instance, wild animals possess more B2 and D strains and fewer A and B1 strains than domestic animals. Herbivores are also distinguished from non-herbivorous animals. Indeed, herbivores have fewer A and more D compared to non-herbivores [15]. Carlos et al. (2010) [14] further collected 241 *E. coli* isolates, and phylo-grouped and sub-grouped them into A_0_, A_1_, B1, B2_2_, B2_3_, D_1_, and D_2._ The composition of the host strains were 94 human strains, 13 chicken strains, 50 cow strains, 16 goat strains, 39 pig strains, and 29 sheep strains, and the prevalence of each of the groups was found as described in Table 1. It is evident from the Carlos study that human strains were predominantly in group B2 (subgroup B2_2_ and B2_3_) with a low abundance of pig strains in B2_2_ (Table 1). 

The specificity of the phylo-grouping approach is dependent on the structure of the *E. coli* population and can vary according to the geographic variation of the *E. coli* population structure [17,21]. The relative abundance of the phylogroups among the hosts can be easily characterized by this approach and can be implemented in different regions of the world as a bacterial source tracking tool. Due to the variability of the host-specific genetic markers to determine the distinction between animal and human fecal sources, in this work, we utilized *E. coli* phylogroups to identify the possible host of fecal contamination in drinking water. 

By analyzing the household drinking water in a low-income urban area in Dhaka, Bangladesh using the phylo-grouping methods developed for environmental samples, this manuscript aimed to investigate the origin of diverse DEC isolates found in the water. The usability of *E. coli* phylogroups as a tool to identify the origin of the DEC and the implications of these findings on future WASH interventions are also discussed.

## 2. Materials and Methods

The study design and water sample collection procedures have been described previously [22,23,24]. 

### 2.1. Study Design

The study was conducted in a low-income urban community located in Arichpur, Dhaka, Bangladesh [23]. Arichpur is a densely populated area with >100,000 residents per km^2^ [23,25]. East Arichpur has a history of outbreaks of waterborne diseases, including cholera [25,26]. The demographic characteristics of East Arichpur have been described elsewhere [23]. East Arichpur had a total of 13,876 households living in 1437 compounds (a cluster of households sharing the same yard and other facilities i.e., water, kitchen, and toilet facilities) [23]. Most (90%) of the households were semi *pacca* (wall made of concrete, roof made of tin or wood), and water collection points were inside the compounds [23]. The floor of the common yard was mostly *Kutcha* (made of mud or brick instead of concrete) [27]. Most (90%) of the compounds had sanitary latrines (with or without a water seal). Most (98%) of families were nuclear families [23]. The formative study findings of the mother study [23] found that the households usually raised pets, poultry, and cattle in their yards. The animals (cows, chickens, pigs, sheep, and goats) referred to in the study by Carlos et al., (2010) in Table 1 were mostly correlated with the domestic animals raised within the premises of study households. To estimate the true water quality experienced by the people in the area, water samples from the ’point-of-drinking’ were collected from the actual drinking utensils: mug, glass, bottle, jug, and pitcher that household members used for drinking. To estimate the public domain contribution to the contamination, water samples were collected from the nearest water taps of the communal source. All of these were connected to one of two different types of water supply systems: (a) a communal WASA (Water Supply and Sewerage Authority) ground water pump installed by the municipal government (138 to 140-m depth), and (b) a number of locally owned ground water pumps operated by individuals or groups of residents. These locally operated pumps are less deep (~85-m depth) compared to WASA and are installed in poorly protected boreholes next to (or in) the streets/footpaths of neighborhood. The WASA communal sources are connected to households through underground network pipes; however, they are often exposed above ground or in gutters/sewage drains, attached to poorly protected and poorly maintained household connections, and the local pumps were connected to overhead polyvinyl chloride (PVC) plastic collection tanks from which the connected households are supplied water through an overground (roof level) PVC piped network. The team selected a total of 477 households from the list of compounds to cohort for 18 months and each household was visited for data and sample collection at six weeks intervals [23]. The detail sampling procedures of households has been described elsewhere [23]. The samples were collected as part of a routine visit at six weeks intervals from September 2014 to October 2015 [22]. As part of this six weeks visit, the team collected 2514 point-of-drinking water samples and 1494 communal sources water samples from September 2014 to October 2015 for water quality assessment [22,24]. From these water samples, 108 point-of-drinking water and 76 communal source water samples were randomly chosen for bacterial isolation. 

### 2.2. Sample Collection and the Culture of Bacterial Strains

Water samples were collected in pre-sterilized wide-mouth water sampling bottles (SPL Life Sciences, Pocheon-si, Korea) and transported in a cool box to the Environmental Microbiology Laboratory, the University of Dhaka, within 2–4 h of collection [24]. Aliquots of 100 mL water samples were filtered through 0.45 µm 47 mm white gridded S-Pak Filters (Merck Millipore, Darmstadt, Germany), and the filters were placed on modified Thermotolerant *E. coli* agar (m-TEC agar, Oxoid, UK) plates. Plates containing the filters were incubated at 44.5 +/− 0.5 °C for 18–24 h. After overnight incubation, typical reddish-purple or magenta colonies on m-TEC were presumptively considered as *E. coli* colonies and enumerated. 

### 2.3. Extraction of Bacterial DNA

Isolates of *E. coli* were routinely grown on a nutrient broth (NB) at 37 °C. Genomic DNA from overnight cultures of *E. coli* strains from NB were extracted using the boiled template method described previously [28].

### 2.4. Detection of Virulence Genes and Phylogenetic Groups

All presumptive *E. coli* isolates were confirmed as *E*. *coli* by real-time PCR detection of the *E*. *coli*-specific housekeeping gene *uidA* [29] and underwent a more extensive virulence gene screen.

*DEC*: Multiplex PCRs with previously published primers (Table S1) were performed to detect the virulence markers of DEC. The criteria for determining the pathotypes of DEC have been described by Nguyen et al., 2011 [30].

*Commensal E. coli*: The *E. coli* isolates, those that tested negative for DEC virulence markers in our PCR assays were defined as commensal *E. coli.*

*Phylogenetic group determination*: The phylogenetic group of each isolate was determined according to Clermont et al. (2000) [20], by a multiplex PCR of the genes *chuA* and *yjaA* and the DNA fragment TspE4.C2 (Table S1). The isolates were assigned to the phylogenetic groups as follows: B2 (*chuA*+, *yjaA*+), D (*chuA*+, *yjaA*-), B1 (*chuA*-, TspE4.C2+), or A (*chuA*-, TspE4.C2). Subgroups within the phylogroups: A_0_, A_1_, B2_2_, B2_3_, D_1_, and D_2_ were determined to increase the distinction among the isolates according to the method described by Escobar-Páramo et al. (2006) [15].

*APEC:* The avian pathogenic *E. coli* (APEC)-associated genes were screened using previously published primers (Table S1).

*PCR product visualization***:** Amplified products were resolved in 1.5% agarose (Carl Roth, Germany) gel using a power pack (Bio-Rad, Hercules, CA, USA) at 80 volts for 45 min. 

### 2.5. Data Analysis

Descriptive statistics were used to analyze the proportions of the pathotypes, and the phylogenetic groups of the *E. coli* isolates. *p* ≤ 0.05 was considered to be statistically significant.

## 3. Results

A total of 228 *E. coli* isolates were obtained in which 125 were isolated from the point-of-drinking water samples, and 103 were isolated from 76 communal source water samples. 

**Figure 1:**
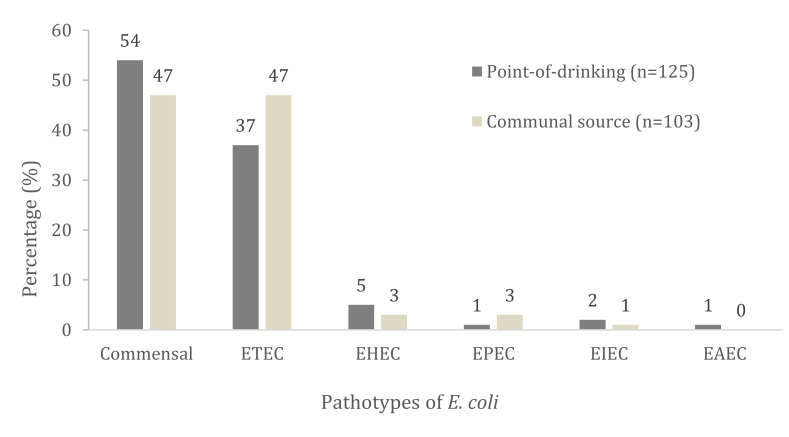
Prevalence of the pathotypes of *E. coli* in source and point-of-drinking water. Calculated among 228 isolates of point-of-drinking and source water in Arichpur, Dhaka collected from September 2014 to October 2015.

DEC was identified in 46% (57/125, CI 95%: 41−58) of the isolates from the point-of-drinking water and 53% (55/103, CI 95%: 45−64) of the isolates from the communal water sources. ETEC was found to be highest among all the DEC isolates, and the communal source had higher ETEC compared to the point-of-drinking water (Figure 1). Of the DEC isolates, 81% (46/57) of the ETEC were found in the point-of-drinking water and 87% (48/55) were found in the source water. The *estA* gene containing ETEC was the most dominant pathotype found both in the point-of-drinking water and the source water (Table 2). EPEC, EHEC, EIEC, and EAEC accounted for 19% (11/57) of the pathogenic *E. coli* isolates in the point-of-drinking water and 13% (7/55) in the source water. 

**Figure 2:**
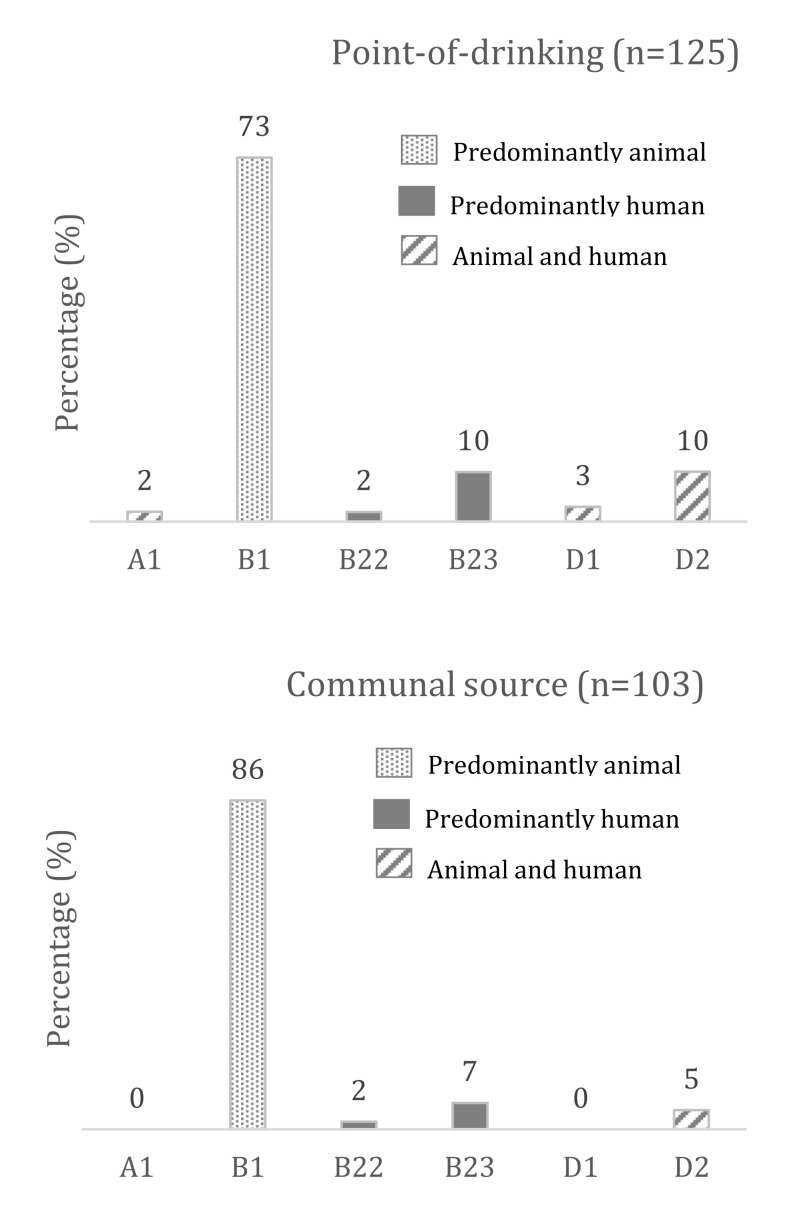
Assignment of phylogroups and subgroups. Phylogroups and subgroups were assigned among 228 isolates from the point-of-drinking water and the source water in Arichpur, Dhaka, collected from September 2014 to October 2015.

Phylogenetic grouping showed that 125 *E. coli* isolates from the point-of-drinking water belonged to six subgroups, and 103 *E. coli* isolates from the public source water belonged to four subgroups (Figure 2). The B1 phylogroup was highest for both the point-of-drinking and the source water isolates, and the differences between the point-of-drinking water and the source water were significant (*p*-value = 0.011^†^) (Table S2). 

Among the DEC pathotypes, ETEC and EPEC belonging to the B1 phylogroup represented the greater percentage of the isolates of the source water compared to the point-of-drinking water (Table 3). On the contrary, a greater percentage of EHEC and EIEC of phylogroup B1 were detected in the point-of-drinking water compared to the source water (Table 3).

APEC associated genes were found in a higher frequency in the point-of-drinking water compared to the source water (Table 4). A significantly higher presence of the *cnf1*, *iss*, and *ibe10* genes was found.

## 4. Discussion

Almost half of all the *E. coli* isolates in the actual drinking vessels were found to be DEC, and the percentage increased to 53% when measured in the water of the communal water source. This suggests that the *E. coli* found in the drinking water in Arichpur should not be seen as a harmless indicator bacterium, but as a potential pathogenic organism. A seven-to-eight-fold higher presence of phylogroup B1 (predominantly animal originated) in both the point-of-drinking water and the communal source water isolates, compared to the presence of B2_3_ and B2_2_ (predominantly human originated) group suggests that fecal contamination originated from non-human mammals and birds, and to a lesser extent from human feces. The wider presence of all six subgroups (A1, B1, B2_2,_ B2_3_, D1, D2), including the presence of human originated subgroups (B2_2,_ B2_3_) in point-of-drinking water compared to source water, indicates that the fecal source of contamination in the point-of-drinking water occurred through diverse host origins in the domestic domain, and a human host potentially played an important role in the inhouse contamination.

The high abundance of the B1 phylogroups from the water samples in our study is consistent with other studies conducted in environmental waters (lakes and rivers) [31,32,33,34]. Stoppe et al. (2014) [16] attempted to identify the main pollution source in rivers and reservoirs water and found that the B1 phylogroup was predominant where exposure to domesticated animal feces was high, and B2_3_ was predominant where exposure to human sewage was high. Moreover, the application of advanced methods such as whole genome sequencing (WGS) found the B1 phylogroup in the rural drinking water of Kenya [35]. Usually, the association between the phylogroup and the strain origin varies based on diet, hygiene, animal domestication status, and morphological and socioeconomic factors [36]. A review of the results from both higher and lower income countries in Europe, Africa, the Americas, Asia, and Australia [36] found that the phylogroup B1 strains were dominant in animals (41%), followed by the A (22%), B2 (21%), and D strains (16%). However, some studies state that strains from B1 and A are better generalists/naturalists that can survive best in the environment [37,38] and are more prevalent in freshwater samples than other strains [39]. Therefore, the dominance of phylogroup B1 in our samples might be due to the fact that it is the naturalist that is better adapted to the environment, and yet the presence of the animal originated *E. coli* cannot be ignored [40]. 

The findings of a wide range of phylogroups with a higher presence of *E. coli* that originated in human feces in the point-of-drinking is also relevant in relation to a study conducted in India, which reported that animal fecal markers were widely detected in both public and domestic domains, and human fecal markers were detected much more frequently in the domestic domain than in public domain sources [8]. 

Our study found a higher presence of the ETEC pathotype consistent with other studies conducted in Bangladesh [41,42,43] and could be of animal origin. Previously ETEC has been found in environmental water in Dhaka and is viable after long-term water incubation [41,44]. A study conducted in an urban slum area of Dhaka showed that ETEC form biofilms in household water tanks/reservoirs throughout the year [45]. Although humans are known to be strict reservoirs for ETEC, a study conducted in Mymensingh, Bangladesh found that among the 35 *E. coli* strains isolated from rectal (livestock) and cloacal (bird) samples, 66% (23/35) carried virulence genes of *E. coli.* Of these virulent *E. coli* strains, 22% were ETEC, and 44% were hybrid STEC-ETEC indicating that ETEC can also originate from animals. 

Among the virulence genes that are more frequently found in the avian pathogenic *E. coli* (APEC) than in the *E. coli* isolates from healthy birds [46,47], are the *iss* (increased serum survival), the *cnf1* (cytotoxic necrotizing factors), and the *ibe10* (brain microvascular endothelium invasion) genes. These genes were found to have a significantly higher prevalence in point-of-drinking water isolates compared to source water isolates. Avian pathogenic *E. coli* (APEC) isolates cause avian colibacillosis [48,49,50] disease in poultry. Therefore, the significantly higher prevalence of virulence genes of avian pathogenic *E. coli* in point-of-drinking water isolates compared to source water indicates poultry contamination of water in the domestic domain. 

A significant positive association between domestic animal husbandry and diarrheal disease in humans was reported by a systematic review in 2014 [51]. The presence of the predominantly animal-originated phylogroup B1 in water samples might result from raising domestic animals and poultry within the household premises. Ercumen et al., 2017 found that animal feces, especially chicken feces, contributed to domestic contamination when comparing households with and without domestic animals in Bangladesh [52]. This is possible as in Bangladesh, poultry roaming within the household premises, including jumping, laying, and brooding eggs inside the living room, kitchen and cooking pots, and using the bedroom as a night shelter for poultry is a common scenario [53,54,55]. Therefore, the ubiquitous presence of animal feces should be given high attention since it also intensifies the risk of exposure to other zoonotic pathogens. 

As groundwater is the source of the communal water supply, it should theoretically be free of contamination. We have, however, seen substantial contamination at the point of collection, i.e., 58% (866/1494) of the samples were found to be contaminated with *E. coli* at an average of 6-250 *E. coli*/100 mL [24], which most likely is due to the unprotected boreholes located within the public areas of the community. These unprotected sources in combination with improper piping might have allowed the intrusion of surface water and subsequently allowed the contamination of a "pure" water source with all the pathogens that can be found in the neighboring environment.

The pathogenic *E. coli* in our study were detected in over 50% of the point-of-drinking and communal source water samples, which is greater than another study conducted in Dhaka, Bangladesh that found 7% of pathogenic *E. coli* in source water samples from the Dhaka municipality [43]. The disparity between the previously reported results and our observed results is possibly due to the incongruence of the chlorine treatment in Arichpur while the municipal water of Dhaka City was chlorine treated by the authority [56].

Altogether, the presence of diverse pathotypes of DEC was higher in the point-of-drinking water compared to the source water. We did not find any report that showed an association between the DEC strains collected from drinking water and their phylogenetic groups. However, similar to the previous studies [57,58,59] that examined the phylogenetic groups of DEC strains of neonatal gut and stool samples, we identified that the majority (88%, 99/113) of the isolated DEC strains from drinking water were also in the phylogenetic group B1 with the greatest number of virulence genes [19,60]. A recent study by Acosta-Dibarrat et al., (2021) investigated DEC from sheep rectal swabs and carcasses and found that almost all the pathotypes (91–100%) belonged to phylogroup B1. Additionally, the ETEC isolates in our study possessed different virulence traits (hybrid isolates of combination from ETEC-EHEC and ETEC-EIEC) and belonged to different phylogenetic groups (B1, B2_3_, D_1_, D_2_) indicating their heterogeneity. These findings suggest that the ETEC strains in this study might have a genetic background that allows the acquisition of virulence factor coding genes of other pathotypes and their adaptability in different ecological niches. 

One of the limitations of our study is that we did not validate the use of the phylo-grouping method by Clermont et al. (2000), which is a method for microbial source tracking in drinking water within the study area by analyzing purposefully selected host species of fecal specimens. Validation is necessary for any microbial source tracking method in each new location to determine whether the selected microbial targets are both present in the analyzed hosts specimens and unique to the intended fecal source in the study area. To the best of our knowledge, there are very limited data on phylogroup distribution among humans and animal reservoirs in this locality. One study by Saha et al. (2021) [61] analyzed the distribution of phylogroups only in poultry farms. Therefore, we could not compare the frequencies from our data with the sourced samples from animals from this country. Hence, this could be a new avenue of work to characterize the source of fecal contamination in this region using this method. Although there are advanced methods such as WGS [35,62,63], micro-array [64,65], and various host specific markers-based techniques [9,10,11,63,66], these methods are costly and are not specific enough for definitive source tracking and therefore demand a further improvement of the methods. 

## 5. Conclusions

Our study findings suggest that non-human mammals and birds played a vital role in the fecal contamination of drinking water. In the recently adopted Sustainable Development Goals (SDGs) to be achieved by 2030, sanitation is primarily focused on the proper management of human fecal matter to reduce the diarrheal burden [67]. In the SDGs, the proper management of fecal matter from domestic animals is largely ignored and less attention has been given to the influence of animal feces on water contamination and diarrheal diseases. Whereas, in line with our study findings, we have discerned the presence of a wider variety of phylogenetic subgroups and more diverse *E. coli* pathotypes in point-of-drinking water compared to source water, implying that the household domain/premise serves as a major cache where both animal and human host sources play a vital role in fecal contamination. Furthermore, the molecular investigation of *E. coli* virulence genes in drinking water has shed light on the fact that the origin of contamination in the point-of-drinking water is not only limited to diarrheagenic diseases, but rather it can also be linked to a zoonotic origin and may prevail in a low-income urban community. Hence, to break the transmission routes of diarrheal pathogens, a ’One Health’ approach would be imperative to employ. This ’One Health’ approach will help to identify potential interventions through a collaborative effort across multi-disciplines (human and veterinary epidemiologists, microbiologists, anthropologists, and other practitioners and policymakers) to achieve the ultimate goal of optimal health outcomes for all. 

## Figures and Tables

**Table 1:** The distribution of phylogenetic groups, subgroups, and host origin based on the published literature.

**Phylogenetic Subgroup by Carlos et al., 2010 [14]**	**Prevalent Host** **Species**	**Carlos et al. [14] (Ssolates, N = 241)**		**Phylogenetic Subgroup by Escobar et al., 2006 [15]**	**Escobar-Páramo et al. [15] (Isolates, N = 2658)**	
		Prevalent	Less prevalent		Prevalent	Less prevalent
**A_0_**	Animal (Carlos et al., 2010, Escobar-Páramo et al., 2006) [14,15]	Animal: 100% (28) [Cow 43% (12/28), chicken 25% (7/28), pig 14% (4/28), sheep 14% (4/28), goat 4% (1/28)]		**A**	Human 43%	Animal 34% [mammals (39%), bird (16%)]
**A_1_**	Humans (Carlos et al., 2010, Escobar-Páramo et al. 2006) [14,15]	Human 61% (38/62)	Pig 27% (17/62), cow 3% (2/62), goat 3% (2/62), chicken 5% (3/62)			
**B1**	Animals, birds (Carlos et al., 2010, Escobar-Páramo et al., 2006) [14,15]	Animal: 88% (71/81) [Cow 36% (29/81), sheep 25% (20/81), goat 16% (13/81), pig 11% (9/81)]	Human 10% (8/81), chicken 2% (2/81)	**B1**	Animal 32% [bird (30%), mammals (32%)]	Human 22%
**B2_2_**	Humans (Carlos et al., 2010, Escobar-Páramo et al., 2006) [14,15]	Human 62% (5/8),	Pig 25% (2/8), chicken 13% (1/8)	**B2**	Human 24%	Animal 13% [bird (20%), mammals (12%)]
**B2_3_**	Humans (Carlos et al., 2010, Escobar-Páramo et al., 2006) [14,15]	Human 100% (7)				
**D_1_**	Animals, birds (Escobar-Páramo et al., 2006) [14,15]	Human 68% (26/38),	Pig 13% (5/38), cow 11% (4/38), sheep 8% (3/38)	**D**	Animal 21% [bird (38%), mammals (18%)]	Human 11%
**D_2_**	Animals, birds (Escobar-Páramo et al., 2006) [14,15]	Human 59% (10/17),	Cow 18% (3/17), pig 12% (2/17), sheep 12% (2/17)			

**Table 2:** Assignment of the pathotypes based on the virulence gene content and the distribution of other extended virulence genes. Pathotypes were assigned among 228 isolates of point-of-drinking and source water in Arichpur, Dhaka collected from September 2014 to October 2015.

Virulence Genes and Pathotypes Assignment	Total no. with Trait n = 228 (%)	Point-of-Drinking Water n = 125 (%)	Source Water n = 103 (%)	*p* Value
***Pathotype assignment (DEC)***				
*eltB*	27 (12)	14 (11)	13 (13)	0.761
*estA*	46 (20)	22 (18)	24 (23)	0.303
*eltB*+*estA*	21 (9)	10 (8)	11 (11)	0.501
**ETEC**	**94 (41)**	**46 (37)**	**48 (47)**	**0.152**
*vt1*	2 (1)	2 (2)	1 (1)	0.896
*vt2*	3 (1)	4 (3)	2(2)	0.112
*vt1*+*eaeA*	1 (0.4)	1 (1)	-	0.361
*vt2*+*eaeA*	-	-	-	-
**EHEC**	**10 (4)**	**7 (5)**	**3 (3)**	**0.152**
*eaeA*	4 (2)	1 (1)	3 (3)	0.231
*eaeA*+*bfp*	-	-	-	-
**EPEC**	**4 (2)**	**1 (1)**	**3 (3)**	**0.231**
*ipaH*	3 (1)	2 (2)	2 (1)	0.672
**EIEC**	**3 (1)**	**2 (2)**	**1 (1)**	**0.672**
pCVD	1 (0.4)	1 (1)	-	0.361
**EAEC**	**1 (0.4)**	**1 (1)**	**-**	**0.361**
**Commensal**	**116 (51)**	**68 (54)**	**48 (47)**	**0.067**

Significance level (*p* ≤ 0.05).

**Table 3:** **Phylogenetic and subgrouping distribution of pathotypes and commensal *E. coli* strains.** Distribution was sought for 228 isolates from the point-of-drinking water and the source water in Arichpur, Dhaka, collected from September 2014 to October 2015.

Categories	Prevalence of Pathotypes by Phylogenetic Group, no. (%)									
	Point-of-Drinking Water (n = 125)						Source Water (n = 103)			
	A_1_ (n = 2)	B1 (n = 91)	B2_2_ (n = 2)	B2_3_ (n = 13)	D_1_ (n = 4)	D_2_ (n = 13)	B1 (n = 89)	B2_2_ (n = 2)	B2_3_ (n = 7)	D_2_ (n = 5)
ETEC (n = 94)	-	36 (38)	-	2 (2)	3 (3)	5 (5)	46 (49)	-	1 (1)	1 (1)
EHEC (n = 10)	-	6 (67)	-	1 (17)	-	-	2 (17)	-	-	1
EPEC (n = 4)	-	1 (25)	-	-	-	-	3 (75)	-	-	-
EIEC (n = 3)	-	2 (67)	-	-	-	-	1 (33)	-	-	-
EAEC (n = 1)	-	1 (100)	-	-	-	-	-	-	-	-
Commensal (n = 116)	2 (1)	45 (40)	2 (2)	10 (8)	1 (1)	8 (5)	37 (34)	2 (2)	6 (5)	3 (3)

**Table 4:** Presence of APEC-associated genes among the 228 *E. coli* isolates from the point-of-drinking water and the source water in Arichpur, Dhaka collected from September 2014 to October 2015.

APEC Associated Virulence Genes	Total no. with Trait n = 228 (%)	Point-of-Drinking Water, n = 125 (%)	Source Water, n = 103 (%)	*p* Value
*iutA*	31 (14)	20 (16)	11 (11)	0.232
*fyuA*	12 (5)	8 (6)	4 (4)	0.388
*cnf1*	30 (13)	24 (19)	6 (6)	0.003 †
*cvaC*	13 (6)	9 (7)	4 (4)	0.275
*Iss*	32 (14)	27 (22)	5 (5)	0.000 †
*ompT*	18 (8)	8 (6)	10 (10)	0.368
*ibe10*	66 (29)	49 (39)	17 (16)	0.000 †

† Significance level (*p* ≤ 0.05).

## Data Availability

Data is contained within the article.

## Supplementary Materials

The following are available online at https://www.mdpi.com/article/10.3390/tropicalmed6040181/s1, Table S1: Primers used in this study for PCR amplification; Table S2: Assignment of phylogroups and subgroups.
