# Supplementary material for: Is It Human or Animal? The Origin of Pathogenic E. coli in the Drinking Water of a Low-Income Urban Community in Bangladesh
Source: Trop Med Infect Dis. 2021 Oct 5;6(4):181. doi: 10.3390/tropicalmed6040181 (PMC8544722; doi:10.3390/tropicalmed6040181)
Supplement: Supplementary file 1 [file tropicalmed-06-00181-s001.zip › tropicalmed-1355315-supplementary.pdf]

Tropical Medicine and Infectious Disease

MDPI

Supplementary Information

Table S1: Primers used in this study for PCR amplification.

Genes	Primer name	Primer sequences (5′→ 3′)	Amplicon size (bp)	References
		**Fecal Detection**		
uidA (real-time PCR)	UAL1939b	atggaatttcgccgattttgc	187	(Heijnen and Medema 2006)
	UAL2105b	attgtttgcctccctgctgc		
		**DEC genes**		
eltB	LT-F	tctctatgtgcatacggagc	322	(Svenungsson et al. 2000)
	LT-R	ccatactgattgccgcaat		
estA	ST-F	gctaaaccagta$^{g}_{a}$ggtcttcaaaa	147	(Svenungsson et al. 2000)
	ST-F	cccggtaca$^{g}_{a}$gcaggattacaaca		
vt1	VT1-F	gaagagtccgtgggattacg	130	(Pollard et al. 1990)
	VT1-R	agcgatgcagctattaataa		
vt2	VT2-F	accgtttttcagatttt$^{g}_{a}$cacata	298	(Svenungsson et al. 2000)
	VT2-R	tacacaggagcagtttcagacagt		
eaeA	eae-F	cacacgaataaactgactaaaatg	376	(Svenungsson et al. 2000)
	eae-R	aaaaacgctgacccgcacctaaat		
bfpA	bfpA-F	ttcttggtgcttgcgtgtctttt	367	(Svenungsson et al. 2000)
	bfpA-R	ttttgtttgttgtatctttgtaa		
ipaH	ipaH-F	gctggaaaaactcagtgcct	424	(Tornieporth et al. 1995)
	ipaH-R	ccagtccgtaaattcattct		
pCVD	EA-F	ctggcgaaagactgtatcat	630	(Schmidt et al. 1995)
	EA-R	caatgtatagaaatccgctgtt		
		**APEC associated genes**		
iutA	iutA-F	ggctggacatcatgggaactgg	302	(Johnson et al. 1997)
	iutA-R	cgtcgggaacgggtagaatcg		
fyuA	fyuA-F	tgattaaccccgcgacgggaa	880	(Johnson and Stell 2000)
	fyuA-R	cgcagtaggcacgatgttgta		
cnf1	cnf1-F	aagatggagtttcctatgcaggag	498	(Yamamoto et al. 1995)
	cnf1-R	cattcagagtcctgccctcattatt		
cvaC	cvaC-F	cacacacaaacgggagctgtt	680	(Johnson and Stell 2000)
	cvaC-R	cttcccgcagcatagttccat		
iss	iss-F	cagcaacccgaaccacttgatg	323	(Johnson et al. 2008)
	iss-R	agcattgccagagcggcagaa		
ompT	ompT-F	tcatcccggaagcctccctcactactat	496	(Morales et al. 2004)
	ompT-R	tagcgtttgctgcactggcttctgatac		
ibe10	ibe10-F	aggcaggtgtgcgccgcgtac	170	(Johnson and Stell 2000)
	ibe10-R	tggtgctccggcaaaccatgc		
		**Phylogenetic**		
chuA	chuA-F	gacgaaccaacggtcaggat	279	(Clermont et al. 2000)
	chuA-R	tgccgccagtaccaaagaca		
yjaA	yjaA-F	tgaagtgtcaggagacgctg	211	(Clermont et al. 2000)
	yjaA-R	atggagaatgcgttcctcaac		
tspE4C2	tspE4C2-F	gagtaatgtcggggcattca	152	(Clermont et al. 2000)
	tspE4C2-R	cgcgccaacaaagtattacg		

Table S2. Assignment of phylogroups and subgroups. Phylogroups and subgroups were assigned among 229 isolates of point-of-drinking and source water, Arichpur, Dhaka collected within the time frame of September, 2014 to October, 2015.

Phylogenetic subgroup	Prevalent host species*	Total no. N=228 (%)	Point-of-drinking water, n=125 (%)	Source water, n=103 (%)	P-value
A_1	Humans (Carlos et al. 2010, Escobar-Páramo et al. 2006, Stoppe et al. 2014)	2 (1)	2 (2)	-	0.195
B1	Non-human mammals, herbivorous animals, birds (Carlos et al. 2010, Escobar-Páramo et al. 2006, Stoppe et al. 2014)	181 (79)	91 (73)	90 (87)	0.011†
$B2_2$	Humans (Carlos et al. 2010, Escobar-Páramo et al. 2006, Stoppe et al. 2014)	4 (2)	2 (2)	2 (2)	0.853
$B2_3$	Humans (Carlos et al. 2010, Escobar-Páramo et al. 2006, Stoppe et al. 2014)	20 (9)	13 (10)	7 (7)	0.327
D_1	Animals, birds (Escobar-Páramo et al. 2006, Stoppe et al. 2014)	4 (2)	4 (3)	-	0.066
D_2	Animals, birds (Escobar-Páramo et al. 2006, Stoppe et al. 2014)	18 (8)	13 (10)	5 (5)	0.117

References

1. Carlos, C., Pires, M.M., Stoppe, N.C., Hachich, E.M., Sato, M.I., Gomes, T.A., Amaral, L.A. and Ottoboni, L.M. (2010) *Escherichia coli* phylogenetic group determination and its application in the identification of the major animal source of fecal contamination. BMC microbiology 10(1), 161.DOI: 10.1186/1471-2180-10-161
2. Clermont, O., Bonacorsi, S. and Bingen, E. (2000) Rapid and simple determination of theEscherichia coli phylogenetic group. Applied and environmental microbiology 66(10), 4555-4558
3. Escobar-Páramo, P., Menac'h, L., Le Gall, T., Amorin, C., Gouriou, S., Picard, B., Skurnik, D. and Denamur, E. (2006) Identification of forces shaping the commensal *Escherichia coli* genetic structure by comparing animal and human isolates. Environmental microbiology 8(11), 1975-1984.DOI: 10.1111/j.1462-2920.2006.01077.x
4. Heijnen, L. and Medema, G. (2006) Quantitative detection of E. coli, E. coli O157 and other shiga toxin producing E. coli in water samples using a culture method combined with real-time PCR. Journal of Water and Health 4(4), 487-498
5. Johnson, J.R., Stapleton, A.E., Russo, T.A., Scheutz, F., Brown, J.J. and Maslow, J.N. (1997) Characteristics and prevalence within serogroup O4 of a J96-like clonal group of uropathogenic Escherichia coli O4: H5 containing the class I and class III alleles of papG. Infection and immunity 65(6), 2153-2159
6. Johnson, J.R. and Stell, A.L. (2000) Extended virulence genotypes of *Escherichia coli* strains from patients with urosepsis in relation to phylogeny and host compromise. The Journal of infectious diseases 181(1), 261-272
7. Johnson, T.J., Wannemuehler, Y.M. and Nolan, L.K. (2008) Evolution of the iss gene in Escherichia coli. Applied and environmental microbiology 74(8), 2360-2369
8. Morales, C., Lee, M.D., Hofacre, C. and Maurer, J.J. (2004) Detection of a novel virulence gene and a Salmonella virulence homologue among Escherichia coli isolated from broiler chickens. Foodbourne Pathogens & Disease 1(3), 160-165
9. Pollard, D., Johnson, W., Lior, H., Tyler, S. and Rozee, K. (1990) Rapid and specific detection of verotoxin genes in Escherichia coli by the polymerase chain reaction. Journal of clinical microbiology 28(3), 540-545

10. Schmidt, H., Knop, C., Franke, S., Aleksic, S., Heesemann, J. and Karch, H. (1995) Development of PCR for screening of enteroaggregative Escherichia coli. Journal of clinical microbiology 33(3), 701-705
11. Stoppe, N.d.C., Silva, J.S., Torres, T.T., Carlos, C., Hachich, E.M., Sato, M.I.Z., Saraiva, A.M. and Ottoboni, L.M.M. (2014) Clustering of water bodies in unpolluted and polluted environments based on Escherichia coli phylogroup abundance using a simple interaction database. Genetics and molecular biology 37(4), 694-701
12. Svenungsson, B., Lagergren, Å., Ekwall, E., Evengård, B., Hedlund, K.O., Kärnell, A., Löfdahl, S., Svensson, L. and Weintraub, A. (2000) Enteropathogens in adult patients with diarrhea and healthy control subjects: a 1-year prospective study in a Swedish clinic for infectious diseases. Clinical Infectious Diseases 30(5), 770-778
13. Tornieporth, N.G., John, J., Salgado, K., de Jesus, P., Latham, E., Melo, M., Gunzburg, S.T. and Riley, L.W. (1995) Differentiation of pathogenic Escherichia coli strains in Brazilian children by PCR. Journal of clinical microbiology 33(5), 1371-1374
14. Yamamoto, S., Terai, A., Yuri, K., Kurazono, H., Takeda, Y. and Yoshida, O. (1995) Detection of urovirulence factors in Escherichia coli by multiplex polymerase chain reaction. FEMS Immunology & Medical Microbiology 12(2), 85-90
